# Concepts for the Integration and Implementation of mHealth Apps for Patients With Mental Disorders: Scoping Review

**DOI:** 10.2196/66340

**Published:** 2025-09-08

**Authors:** Felix Plescher, Klemens Höfer, Jürgen Wasem, Anna Bußmann, Stefanie Solar, Michael Minor, Sarah Schlierenkamp, Dieter Best, Josepha Katzmann, Enno Maaß, Udo Schneider, Anja Wadeck, Sophia Zander, Carina Abels

**Affiliations:** 1 Institute for Health Care Management and Research University of Duisburg-Essen Essen Germany; 2 Essen Institute for Health Care Management and Research (EsFoMed) Essen Germany; 3 German Psychotherapists Association (DPtV) Berlin Germany; 4 Techniker Krankenkasse Hamburg Germany

**Keywords:** digital health, integration, implementation, mental disorders, mental illness, mobile health, mobile health apps, mHealth apps, Digitale Gesundheitsanwendungen, DiGA, scoping review

## Abstract

**Background:**

Mental and behavioral disorders affect approximately 28% of the adult population in Germany per year, with treatment being provided through a diverse health care system. Yet there are access and capacity problems in outpatient mental health care. One innovation that could help reduce these barriers and improve the current state of care is the use of mobile health (mHealth) apps, known in Germany as Digitale Gesundheitsanwendungen (DiGA). DiGA are certified apps reimbursed by statutory health insurance funds. They are evaluated before use, for example, by proving their medical benefit in clinical studies, or approved for use according to the concept of “coverage with evidence development.” Most DiGA currently included in the DiGA directory are approved for use by people with mental disorders. However, the low and stagnant use numbers point to barriers to their uptake and integration in the German health care system. The analysis of concepts for implementation and integration is important to find effective ways to realize the potential of these apps in mental health care.

**Objective:**

This scoping review aimed to identify concepts and frameworks for the successful integration of mHealth apps into international health care systems and how they can be adapted and transferred to DiGA used in the German health care system.

**Methods:**

The scoping review followed the methodological framework for scoping reviews proposed by Arksey and O’Malley. The scientific databases PubMed, Embase, and PsycINFO were searched. A 2-stage (title and abstract, followed by full text) screening process was conducted by 2 different reviewers independently.

**Results:**

A total of 3710 publications were identified. Of these, 20 were included in the review, with 3 additional studies identified through a targeted search. Results are presented in accordance with the PRISMA-ScR (Preferred Reporting Items for Systematic Reviews and Meta-Analyses Extension for Scoping Reviews) checklist. The literature identified through this scoping review provides an overview of a variety of aspects of mHealth apps for mental disorders and their integration into health care systems. There are different general, organizational, legal, and technical requirements to create a suitable environment, therefore specific concepts for integration and implementation of mHealth apps are necessary. We identified regulatory concepts, for example, directories for mHealth apps, and care-related concepts, for example, training and support of medical professionals. The generated evidence contributes to improved and user-oriented structures and processes, potentially leading to better patient support through mHealth apps.

**Conclusions:**

Evidence already shows that mHealth apps can effectively support patients with mental health disorders. To realize this potential, mHealth apps need to work within the structures and processes of health care systems. Therefore, this scoping review provides insights on the integration of mHealth apps into (outpatient) mental health care.

## Introduction

### Background

Mental disorders annually affect approximately 28% (approximately 17.8 million people) of the adult population in Germany. The most common conditions include anxiety disorders, mood disorders, and substance use disorders [1]. For those affected and their relatives, mental disorders represent a substantial personal and emotional burden. In addition, mental disorders are associated with sociodemographic challenges, such as extended periods of work absence. One German health insurance fund states that from 2022 to 2023, there was an increase in work absence due to mental disorders of >20% for both men and women: the largest increase among all diagnosis groups [2].

To meet this demand, an extensive help and health care system for people with mental disorders exists in Germany. Depending on clinical factors, symptom severity, course of illness, and patient preferences, a range of low-threshold psychosocial interventions, as well as medical, psychotherapeutic, and other treatment options are available [3].

However, there are challenges regarding access and capacity of (outpatient) mental health care: patients are confronted with waiting times before starting treatment, possibly leading to worsening or chronification of diseases [4]. A large share of patients is exclusively treated by general practitioners or specialists in somatic medicine, without ever receiving treatment by psychotherapeutic or psychiatric specialists [5].

A new, innovative low-threshold approach addressing these problems is provided through mobile health (mHealth) apps. They possess the potential of improving access to care and providing assistance to people experiencing mental disorders, bridging waiting times until guideline therapies and supporting psychotherapeutic and psychiatric treatment processes [6]. The German “Council of experts for the assessment of developments in the health care system” confirmed in their report “Digitalization for Health 2021” that mHealth apps have not become a sufficiently used part of health care provision in Germany yet. However, a responsible and scientific uptake of digitization potentials could improve health care support for people in Germany [7].

With the goal of using the potentials of digital technologies for statutory insured people, the German government introduced mHealth apps (called Digitale Gesundheitsanwendungen [DiGA] in Germany) to the German health care system [8]. Since the Digital Healthcare Act came into force on December 19, 2019, mHealth apps are officially part of services reimbursed by the statutory health insurance (SHI) funds. To be implemented into the benefits catalog of the German SHI, mHealth apps need to prove clinical efficiency and patient safety through an evaluation and certification procedure [9]. Physicians can prescribe mHealth apps listed in the DiGA directory, which is being curated by the Federal Institute for Drugs and Medical Devices (BfArM). Patients can also get DiGA directly (without a physician prescription) from their SHI fund, if the corresponding indication is diagnosed and documented by a health care professional. The mandatory review process for a listing in the DiGA directory is called DiGA Fast Track, which is conducted by the BfArM. During this process evidence of a medical benefit or other, patient-relevant structural or procedural improvements (eg, improvement of adherence) and additional general requirements such as data security or interoperability must be provided. DiGA can either provide all evidence upon application for the listing in the DiGA directory to be reimbursed or receive a provisional approval, which requires evidence to be generated within a period of up to 2 years while already being reimbursed by the SHI (coverage with evidence generation) [10].

DiGA for mental disorders represent the most common indication of apps listed in the DiGA directory, with 26 out of 55 listed apps as of September 2024 (excluding DiGA, which have been removed from the DiGA directory) [11]. Use of DiGA has been off to a slow start, and prescription numbers are just slowly increasing. By September 2021, only approximately 50,000 DiGA were prescribed [12]. This number rose to approximately 200,000 by September 2022 and 370,000 by the end of 2023 [13]. Despite this growth, DiGA still represent a relatively small part of the German health care system [13]. Research has shown that barriers regarding the use of DiGA include poor usability, concerns personal contact with health care professionals may be reduced, and a lack of customization to individual needs [14].

### Objectives

To realize the potentials of mHealth apps and improve care and support for patients with mental disorders, the lack of integration into existing care structures and processes—as, for example, described by Giebel et al [15]—has to be considered. International evidence on integration and implementation of mHealth apps into outpatient mental health care systems can provide guidance to improve the German approach. Therefore, we aimed to map the international evidence on integration and implementation of mHealth apps into health care settings exploratively. This decision was made for 2 main reasons: first, the definition of mHealth apps used within this review was based on the concept of DiGA, as described earlier (DiGA differ from other mHealth apps, eg, in terms of the approval process or the different reimbursement options). Second, other research (eg, [15]) suggested that the integration of mHealth apps (in this case also based on DiGA) into health care systems has to be considered to improve this health care service. To the best of our knowledge, the described research rationale has not been systematically studied. We decided to address this research gap using the scoping review approach.

More information about the associated research project can be found in the published study protocol [16].

## Methods

### Overview

This scoping review aimed to provide an overview of key processes, structures, and other aspects to consider, which are necessary to integrate mHealth apps into existing mental health care contexts sufficiently. This can be achieved in two ways: (1) already existing mHealth apps can be integrated into established health care contexts, for example, by monitoring mHealth app use, and (2) new interventions can be implemented in existing mental health care contexts. Both concepts, integration and implementation, are the objectives of this review.

This scoping review is guided by the framework proposed by Arksey and O’Malley [17], with refinements suggested by Levac et al [18]. The PRISMA-ScR (Preferred Reporting Items for Systematic Reviews and Meta-Analyses Extension for Scoping Reviews) checklist will be used for reporting the results and the final manuscript [19]. This approach was chosen because we wanted to identify and summarize in how far the existing literature can be used to enhance the integration of DiGA into health care services. This is in alignment with the purposes for scoping studies by Arksey and O’Malley [17], for example, “to summarize and disseminate research findings.” The enhancement by Levac et al [18] was also incorporated, as their approach is based on the framework proposed by Arksey and O’Malley [17] and provides more detailed guidance for every framework stage.

Throughout this publication, the term “mHealth apps” is used for all mHealth apps meeting our definition laid out in the eligibility criteria, including digital health applications (DiGA). The short form DiGA will be used if the corresponding passage is only about the German concept of DiGA.

### Step 1: Identifying the Research Question

The goal of this review is to identify frameworks and concepts for integration and implementation of mHealth apps into processes and structures of mental health care systems. The following research question will be answered throughout this review: “Which concepts for integrating or implementing mHealth apps into care for people with mental disorders exist globally and how can they be transferred to the German health care system?”

To develop the search strategy, the research question was translated into the population, concept, and context (PCC) framework [20]. We used the PCC framework instead of the population, intervention, control, and outcome (PICO) framework because our research question does not aim at a specific outcome or comparison intervention. For this purpose, the PCC framework is recommended by the JBI Manual for Evidence Synthesis [21].

The PCC framework translation used in this study is displayed in Textbox 1 [21].

**Population, concept, and context (PCC) framework.**

- Population: patients with mental disorders
- Concept: mobile health apps
- Context: integration into health care systems

We conducted an explorative search to identify keywords that could be used to operationalize the described PCC framework. In addition, we used the thesauruses of the searched databases to add equivalent and not-yet-included keywords as well as mapping terms, such as Medical Subject Headings (MeSH) terms. The identification of concepts, frameworks, and other evidence regarding the integration and implementation of mHealth apps was operationalized within the inclusion and exclusion criteria.

### Step 2: Identifying Relevant Studies

The search was conducted on January 25, 2023 using the PubMed, Embase, and PsycINFO databases. We used the 3 components of the PCC framework and combined them with the Boolean “AND” operator to create our search string. Keywords within each search block were combined using the Boolean “OR” operator. In addition, all search strings were adjusted to database specifics. The search string used for PubMed (MeSH terms are not presented here, see Multimedia Appendix 1) is as follows: (mental disorder* OR mental illness* OR mental disease* OR mental health OR psychiatric disease* OR psychiatric disorder*) AND (implementation* OR integration* OR introduction OR adoption OR insertion OR framework OR guideline* OR strateg*) AND (digital health app* OR mHealth OR eHealth OR healthcare app* OR health care app* OR mobile health OR health app* OR virtual care OR digital intervention OR web app* OR mobile app* OR smartphone OR smart phone OR mobile phone OR android OR iphone OR browser).

Only titles and abstracts that included these individual search terms were considered. Furthermore, MeSH terms were added to expand the search terms. A more detailed version of the conducted search can be found in Multimedia Appendix 1.

### Step 3: Study Selection and Eligibility Criteria

The eligibility criteria used for study selection are described in Textbox 2. We selected them after consulting other reviews covering mHealth apps and adapted them according to our research goal.

The BfArM describes mHealth apps (in Germany: DiGA) as a medical device, whose medical purpose is achieved primarily by the digital technology of the app. DiGA assist in the detection, monitoring, or treatment of the disease and require interaction of the patient with the DiGA [8]. Germany has a high standard concerning data privacy requirements, which mHealth apps have to fulfill to be considered a reimbursable DiGA. On the one hand, as there is no uniform definition for mHealth apps internationally, we decided to explicitly define the excluded apps (see Textbox 2: “lifestyle apps”), as they are not comparable to the German DiGA equivalent. We specifically designed the definition to capture and exclude all apps that do not meet the DiGA criteria. On the other hand, we left room to include mHealth apps exceeding the definition of DiGA as of today, as the legal definition of DiGA might be extended in the future. This may lead to the introduction of, for example, higher risk classes according to the Medical Device Regulation [27,28]. Therefore, we ensured that we include mHealth apps meeting DiGA criteria as of today and—if adaptions and extensions to the legal definition are made—in the future.

The eligibility criteria were used by 2 independent researchers (S Solar and FP) for the screening process of the identified articles. First, all titles and abstracts were evaluated, followed by a full-text screening. A numerical description of the studies excluded at corresponding screening step can be found in the section Selection of Evidence.

**Inclusion and exclusion criteria (based on previous studies [9,22-25]).**

**Inclusion criteria**

- Concepts for integration or implementation of mobile health (mHealth) apps into the care of patients with mental disorders include the following: description or testing of structural requirements for the integration of mHealth apps (eg, participating institutions, actors, and infrastructure); description or testing of processes for the integration of mHealth apps (eg, design of prescription processes and monitoring processes); combinations of the aforementioned points
- The publication or the included apps are used in the context of mental disorders (F-diagnoses International Classification of Diseases, Tenth Revision, [ICD-10])
- The apps examined do not meet the definition of a lifestyle app
- Outpatients or inpatients who switch to the outpatient sector (aftercare and discharge management)
- Abstract and full text available
- Age group: ≥12 years
- Articles written in German or English
- Publication period: from 2007 (introduction of the iPhone [26]) to the end of 2022

**Exclusion criteria**

- The publication does not deal with either of the described main topics
- The publication or the included apps are not used in the context of mental disorders (F-diagnoses [ICD-10])
- The apps examined correspond to ≥1 criteria of the following definition of lifestyle apps: not related to the diagnosis, cure, mitigation, or treatment of (mental) disorders or health conditions; designed for primary prevention and used by healthy users who have not been diagnosed with a disease or have no (documented) indication; used exclusively by physicians without active patient involvement; only aim at enhancing motivation and are intended to maintain or encourage a healthy lifestyle; used exclusively as a communication medium and are “telemedical” apps (pure communication tools) between the patient and physician; used exclusively for data collection and do not “use” the collected data; do not meet the requirements for medical devices (eg, Medical Device Regulation and United States Food and Drug Administration regulation); are not certified as a medical device (if used within the European Union); and have a higher, nonadmissible risk class
- Only inpatients admitted
- Research protocol or conference abstract; abstract or full text not available
- Age group: <12 years
- Articles written in another language than English or German
- Publications before or after the publication period

### Step 4: Charting the Data

After the screening procedure, the eligible studies were included. The information from the literature was charted and quality assured by 2 researchers. This included assessing general information about the publication, the population studied, the methods used, and the corresponding mHealth app or the concept used to integrate them. The information about the included studies can be found in Multimedia Appendix 2.

### Step 5: Collating, Summarizing, and Reporting the Results

According to the framework extension by Levac et al [18], we followed 3 substeps to present our results: we analyzed the data, including numerical summaries of the information (eg, the overall number of studies included and types of study design). In this step, we used software supported qualitative thematic analysis techniques [29] to further extend the numerical analysis of the data. The included studies were analyzed using both deductive and inductive codes. For this purpose, we used MAXQDA (VERBI GmbH) [30], an established software program for qualitative research that is familiar to all members of the research team. The initial coding scheme included descriptive information about the included studies (1=study, 2=population, 3=methods, and 4=mHealth app) and 1 category for evidence regarding the research question (5=concepts for integration and implementation). Subcodes were developed in a 2-step approach. First, 2 authors (FP and S Solar) analyzed the studies using the 5 main categories. The evidence within each main category were sorted into groups, representing subcodes (1.1, 1.2, 1.3...). Second, the subcodes were compared between both authors. Matching subcodes were added to the coding scheme. The remaining subcodes were discussed. In case of disagreement, senior researchers (CA and AB) were consulted. After resolving all points of discussion, the coding scheme was adapted and finalized. The final coding scheme was used for all included studies and can be found in Multimedia Appendix 1*.* This was followed by the narrative report of the results specifically aimed at answering the research question. Afterward, these results were discussed in a broader context. For this, research covering similar topics, recent policy advancements, the practice perspective, and the political discourse were considered [18].

### Step 6: Targeted Literature Search

Going along with the methodological framework of scoping reviews by Arksey and O’Malley [17], we carried out an additional targeted literature search to identify relevant articles not included by the systematic search. As our research question also aimed at evaluating the translation of concepts to the German health care system, we primarily wanted to ensure that all concepts for integration and implementation already established in Germany were included. Therefore, the targeted literature search focused on the German health care system.

The identification of the literature was carried out through a 2-step approach. First, we created a list of all relevant stakeholders. Second, we searched through websites and digital archives using the search terms from the systematic search (refer to Identifying Relevant Studies section). The same eligibility criteria were also used for the selection of relevant studies.

The synthesis of results will present both the evidence from the targeted search and the systematic review.

## Results

### Selection of Evidence

**Figure 1:**
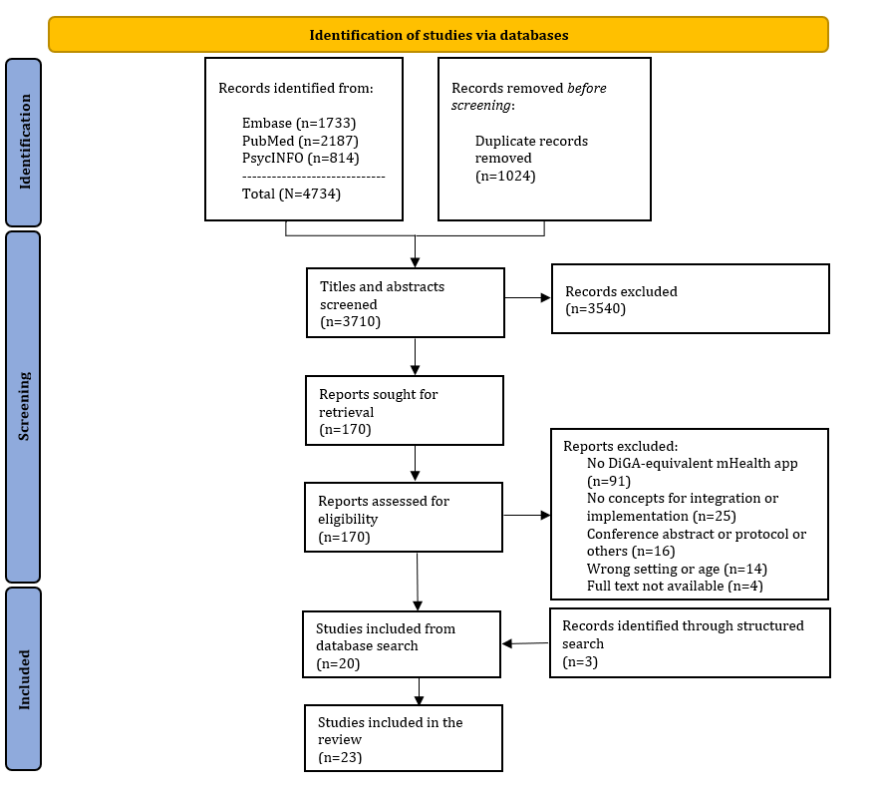
Flow diagram: selection of sources of evidence.

Of 4734 records, 3710 (78.37%) were identified through the systematic search, and 1024 (21.63%) duplicates were removed. Title and abstract were screened, and of the 3710 articles, 3540 (95.42%) were excluded, leaving 170 (4.58%) articles for full-text screening, with 20 (0.54%) articles fulfilling all inclusion criteria (refer to Figure 1 and Multimedia Appendix 3 for the excluded studies) [31-50]. In addition, 3 articles [51-53] were identified through the targeted literature search. In the end, 23 articles were included in the final review.

### Included Studies

Of the 23 included studies, 9 (39%) used qualitative methods [31,34,36,37,39-41,43,48]. Other studies used quantitative methods (1/23, 4%) [44], mixed methods design (5/23, 22%) [32,38,45-47], or literature reviews (1/23, 4%) [33]. Furthermore, 30% (7/23) of the studies—also including studies from the targeted literature search—were nonsystematic narrative reviews [35,42,49-53]. All the qualitative and mixed methods studies used interviews or focus groups with either patients or medical professionals [31,32,34,36-41,43,45-48]. The quantitative study used surveys to identify both patients and medical professionals’ perspectives [44]. One metareview was included, which provided an overview of 9 systematic reviews [33].

The most studied mental disorder throughout the identified records was depression. Of the 23 studies, 4 (17%) investigated mHealth app use for minor to severe depression [33,34,37,48]. Other indications were psychosis [31,45]; personality disorders [32]; mood and anxiety disorders [36]; bipolar disorders [38,39]; substance use disorders [40]; drug use disorders [41,43]; schizophrenia [44]; insomnia [46]; posttraumatic stress disorder (PTSD) [47]; anxiety [48]; and broad terms, for example, “mental health conditions” [35], “patients with mental health diseases” [42], or “mental disorders” [49,50].

There were differences between the studies regarding the included apps. Some studies exclusively looked at certain mHealth apps, for example, “A-Chess” [41] or “CBT-I Coach” [46]. Other studies with a broader perspective used the concept of mHealth apps as an objective. This was especially the case for studies that looked at the German health care context and therefore at the concept of DiGA [42,49].

The evidence mostly originated from North America (United States: 10/23, 43%; Canada: 1/23, 4%) [35-37,40,41,43-48] and Europe (7/23, 30%) [31,32,38,39,42,49,50]. Three studies involved research from multiple countries [31,34,45]. Germany made up the largest share of European studies (4/23, 17%) [39,42,49,50]. The United Kingdom [31], Denmark [32], and Ireland [38] were each represented in 4% (1/23) of the studies. The remaining studies originated from Australia (4/23, 17%) [31,33,34,45] and New Zealand (1/23, 4%) [34].

### Synthesis of Evidence

#### Overview

The presented information is divided into 2 parts. First, general conditions for integrating mHealth apps into health care systems focusing on outpatient mental health care are described. This is followed by concepts for integration, which are presented covering theoretical, regulatory, and care-related aspects. We derived these categories from the coding scheme mentioned above (refer to Step 5: Collating, Summarizing, and Reporting the Results section). The coding scheme was further aggregated into the final categories presented in Textbox 3. An overview with descriptions for each category is provided in Multimedia Appendix 4.

**Categories used for evidence synthesis.**

**General requirements**

- Financial considerations
- Legal considerations
- Organizational considerations
- Technological infrastructure

**Concepts for integration**

- Theoretical aspects
- Regulatory concepts: evaluation of mobile health apps; medical training and curricula; directories
- Care-related concepts: adherence-improving strategies; clinical workflow and adaptation; guided app use; integration of additional technical components; public relations and information; training and support of users and medical professionals

#### General Requirements

##### Overview

To integrate mHealth apps into health care contexts, certain requirements have to be met to enable a successful and sustainable integration. Across the identified studies, different thematical focuses emerged, which we grouped into organizational, financial, legal, and technological requirements. A large share of the included studies addressed these topics [32,33,40-42,45,47-50], which are presented in the subsequent sections.

##### Financial Considerations

As all services provided within a health care system trigger different types of costs, financial requirements and their underlying regulatory aspects must be addressed. This includes funding, reimbursement, and the provision of services carried out by medical professionals, which was discussed in 4 studies [37,41,42,49]. Paying for mHealth apps and therefore ensuring that patients can use them free of charge was seen as “critical for long-term use, primarily in under resourced communities” by Dinkel et al [37]. In most health care and insurance systems worldwide, this requires funding from health insurance funds [42]. For example, in Germany, the use of mHealth apps by patients is already reimbursed [42,49]. However, it is not just the apps themselves that need to be reimbursed. According to Gerlinger et al [42], all services and actions carried out by medical professionals surrounding the use of the mHealth app need to be financed as well, ensuring that the financial burden of use and integration of these mHealth apps is not exclusively carried by the medical professionals.

This was also reported to be the case for health care systems not organized on a federal level. Ford et al [41] also pointed out that funding, often in close dialogue with health insurance companies, is crucial.

##### Legal Considerations

Introducing new services into the benefits catalog also requires a clear definition of the legal and judicial conditions, which was addressed in 1 study [36]. This includes the definition and borders of liability of manufacturers and medical professionals in the context of mHealth apps. The study highlighted the importance of a clear definition of (legal) responsibilities of medical professionals, especially because mHealth apps are available to the patients at any time [36].

##### Organizational Considerations

Organizational requirements were described in 6 studies [36,41,42,47-49]. On an organizational level, support from leadership and their engagement was reported as key to successful integration of mHealth apps according to 3 studies [41,47,48].

In addition, 3 studies pointed out that sufficient organizational processes and structures are essential when integrating mHealth apps into any health care system. Therefore, regulatory oversight was mentioned to be important for ensuring and providing the necessary infrastructure [36,41,42].

The included studies also highlighted the importance of prescription of mHealth apps by medical professionals [42,49].

##### Technological Infrastructure

A total of 4 studies highlighted technological requirements for optimal integration of mHealth apps into health care systems [32,40,42,43]. These requirements are supposed to ensure patients can use mHealth apps and the corresponding processes “seamless and free of technical glitches and other barriers that could decrease their motivation or lead to ‘getting stuck’” [43]. Two studies proposed that a superordinate, technological infrastructure is needed to ensure a safe, reliable, and standardized operation of mHealth apps [32,42]. Two studies added that in case of problems, technical support for all user groups must be available [32,40]. One study stated that the access to necessary equipment (eg, internet access and computers) needed for use should also be provided according to user preferences when integrating apps [40].

#### Concepts for Integration

##### Overview

Integration and implementation of mHealth apps was facilitated through a variety of concepts and strategies. The extracted evidence provided conceptional basics and models to consider for integration and a set of different strategies [32-50].

We further organized the identified concepts of integration into theoretical or conceptional, regulatory, and care-oriented aspects.

##### Theoretical Aspects

Theoretical concepts and models, which can be used to guide and facilitate mHealth app integration were addressed in 4 studies [33,42,46,50]. Different concepts were presented focusing on mHealth apps, while others targeted the implementation and integration process itself.

The study by Possemato et al [45] used the Consolidated Framework for Implementation Research, which is frequently used for implementation in research and covers the organizational side while also considering patient factors. The five main domains of the construct are (1) the intervention of interest, (2) the inner setting, (3) the outer setting, (4) the individuals involved, and (5) the process of implementation. Each domain includes constructs that influence implementation. Possemato et al [45] used the framework to identify the constructs most important for providers when implementing a guided mHealth app, the coach-supported “PTSD Coach,” an intervention for patients with PTSD. The study shows that, for example, the compatibility with the primary care setting, patient engagement, or knowledge and beliefs about the intervention are important constructs from a provider perspective when implementing mHealth apps into health care settings [47].

##### Regulatory Concepts

#### Overview

A total of 7 studies stated regulatory processes as a fundamental requirement for integration [35-37,42,48-50]. Two studies generally stated that federal programs or government policy are necessary to increase access and ensure structurally sufficient embedding of mHealth apps [36,37].

In addition, regulatory initiatives such as the systematic evaluation of mHealth apps were addressed. These are presented in the subsequent sections, including evaluation, medical training and curricula, and directories.

#### Evaluation of mHealth Apps

Ensuring medical benefits and safety for patients was covered by 4 studies reporting on the evaluation of mHealth apps [35,42,48,50]. A range of established evaluation models was mentioned, including the American Psychological Association pyramid model, Elight, and the Mobile App Rating Scale [35].

As 1 of the first countries worldwide, Germany implemented a screening process for mHealth apps, the so-called “DiGA Fast Track,” as described above (refer to Background section) [49].

In a country where a federal evaluation process (DiGA Fast Track) already exists, additional evaluation initiatives are also used. Independent evaluations, without involvement of the app manufacturer, in addition to a federal process were called for by Gerlinger et al [42]*.* The concepts of “APPKRI,” developed by the Fraunhofer Institute, or the app quality concept “AppQ” by the Bertelsmann Foundation aimed at evaluating mHealth apps independently from the DiGA Fast Track. “AppQ” was further developed within a project called “Weisse Liste” (whitelist), which ranked mHealth apps according to their quality scores [42].

#### Medical Training and Curricula

Gerlinger et al [42] proposed the integration of mHealth apps (in the German context: DiGA) into medical training of medical professionals and further education throughout the professional career, with sufficient funding and reimbursement for these efforts.

#### Directories

Closely linked to the systematic evaluation of apps are directories, which provide an overview of the apps with corresponding information. Particularly when having to choose between different apps, well-designed portals can guide medical professionals as well as patients through the large number of mHealth apps, which was addressed in 1 study [49].

In Germany, the DiGA directory provides information about all preliminary and permanently accepted mHealth apps (in this case DiGA) [9]. In addition, other portals have been developed to transparently inform about DiGA. The project “Weisse Liste” developed their own directory “App-Search” based on the AppQ evaluation scheme (refer to Evaluation of mHealth apps section). Other examples are the “Mobile Health App Database” or “psychenet” [49].

##### Care-Related Concepts

#### Adherence-Improving Strategies

As part of integration and enhanced use of mHealth apps into care, 7 studies described strategies aiming to improve adherence of patients using mHealth apps [37,40-44,48]. As a lot of mHealth apps use monitoring and self-assessment features, sufficient adherence is necessary to produce reliable data and results, according to these studies [37,40-44,48].

The studies addressed various examples for direct adherence enhancement, such as automized reminders [37,43,44], chat support [37], and monitoring as well as notifications of app use [41,48]. An alternatively mentioned approach to increase the app use is by indirectly motivating patients and users through incentives. Gift certificates or milestones within the app to motivate patients to increase or keep up app use were reported by 2 studies [37,40].

#### Clinical Workflows and Adaption

When integrating mHealth apps into the health care context, clinical workflows must be reviewed and adapted, if necessary, which was addressed in 10 of the identified studies [33,35-37,40,41,43,47,48,50]. According to Enrique et al [38], processes and tasks involving the integration of mHealth apps have to be perceived with the same value as other “traditional” clinical tasks. They added that when creating and adopting workflows, the perspective of the involved stakeholders is a key requirement [38]. Another common problem that was highlighted in the studies is the work capacity and time availability. Integration of mHealth apps and the associated tasks need to fit into the regular workday rather than “on top” and thus creating an additional burden [47]. To address this, 1 study proposed that it might be necessary to rethink current processes and reduce workload to make room for tasks involving mHealth apps and their integration [40].

One way to adapt workflows and also consider the unavailability of work capacity according to the identified studies is integrating screening tools into, for example, anamnesis. A study about a coach-supported mHealth app for PTSD (CS-PTSD Coach) described sessions to guide app use, which are further described below (refer to Guided App Use section). One of these sessions was used as orientation and introduction, including PTSD symptom assessment (eg, PTSD Checklists) [47]. In the German context, Gerlinger et al [42] suggested considering digital competencies of patients, prior use of DiGA, or specific DiGA risks to align anamnesis with the increasing task of mHealth app prescription [42].

#### Guided App Use

One strategy to integrate mHealth apps into care is integrating medical professionals into the processes surrounding the use of these apps—so-called guided app use—which was reported in 7 studies [32,39,42,43,47,49,50]. As Fassbinder et al [39] pointed out, the aspiration of a guided model is to monitor the use of the corresponding tool, check if patients use them correctly, and help with problems arising throughout the process. This can lead to increased adherence, as, for example, described by Kreyenbuhl et al [44], and integration of digital treatment into the general treatment process [54]. It can be further distinguished between a guided app use, which is focusing on ensuring that the patient is actually using the app, and a more invasive way by integrating the generated data into the traditional treatment process.

To generate data that can be integrated into treatment, patients document data within the mHealth app themselves, which was reported by 2 studies. They mentioned that the parameters are then transferred to the medical professional and discussed and therefore can enhance measurement-based care [41,44]. The studies also described monitoring the use as an approach to guide the patient [39].

A way to combine clinical standard care and digital solutions are blended care models, an evidence-based approach for integration of mHealth apps into health care contexts, described by Gerlinger et al [42].

These interventions intertwine face-to-face treatment and, for example, mHealth apps. According to 2 studies, the digital component takes an active part in these treatment approaches by, for example, teaching psychoeducational content [47,49]. One study provided an example of an mHealth app called “PTSD Coach,” which was blended together with in-person visits in a low-intensity stepped-care intervention [47].

Some studies also provided protocols of what guided app use could look like and how different sessions were structured. These often-included tasks before, during, and after app use [43,47].

In a study about an mHealth app for treating patients with drug use disorder, an approach—based on participant (in this case, adult patients) preferences—was described as follows: the first stage introduced the app to the patient and provided information as part of an in-person visit. In the second stage, referred to as the “setup stage,” patients received help with setting up the app, which was partly preferred to be within in-person visits but also over the phone. The third stage involved follow-up and support through phone calls or direct messages to keep patients engaged with the app [43].

Another protocol for guided app use was proposed by Possemato et al [47]. Their intervention “CS-PTSD Coach” consists of 4 sessions and incorporates support at multiple stages: before the app is used (goal setting), during use (facilitation of app use), and after or toward the end of use (follow-up to reinforce the ongoing treatment plan). Similar to the process described earlier, only the first session occurred in person. In accordance to the preferences of the patient, the remaining sessions can be conducted either by phone or in person [47].

Glass et al [43] made a point about guided app use for sensitive diagnosis, highlighting the importance of establishing a trust scheme. As they reported, only trusted people (eg, a therapist consulted before app use) should be involved in guided app use to minimize the burden for patients of having to talk to someone unfamiliar about sensitive topics [43].

#### Integration of Additional Technical Components

The integration and connection of mHealth apps with or within other technical (and telemedical) components was mentioned in 5 studies [37,41,42,44,48]. The main focus was reported to be transferring the generated data out of the mHealth app and integrating it into external platforms. In 1 study, the counselors had access to an external tool, which allowed them to access the patients’ data [44]. Another proposed solution was the transfer of mHealth app–generated data into electronic health records [37,44,48] with similar functionality, such as tracking individual patient progress or disease-related scores [44,48]. One study proposed a platform for all available telemedicine solutions within the health care system [42].

The identified literature also included interaction on these platforms initiated by the patients to the medical professionals and vice versa. Ford et al [41] described the possibility for clinicians to reach out to patients via private messaging, while Kreyenbuhl et al [44] reported the possibility for patients to request to talk about aspects regarding app use.

#### Public Relations and Information

Public relations and information initiatives were mentioned in 6 studies, addressing medical professionals and patients at the same time [33,37,40,41,43,46]. Most studies described this as a problem and also provided solutions on how to solve this and improve the information deficit surrounding mHealth apps. Some initiatives within the included literature focused on designing information material, such as flyers (eg, brochure for RISE Iowa app) [40]. Others focused on increasing public relations by going to community-based gatherings [41] or enhancing the marketing of the apps [46].

#### Training and Support of Users and Medical Professionals

Training and support of patients using the app as well as medical professionals was mentioned in 16 studies [32-36,38,40-43,45-50]. At the beginning of the treatment, multiple studies suggested introducing the app to the patient and teaching important tasks, for example, in the so-called “onboarding” processes [34]. This support was described to be further extended to the period during mHealth app use to assure long-term success and engagement [34,38,43]. In addition, information material, which can be handed out to the patient, can help patients dealing with minor problems, according to Fledderman et al [40]. This is particularly important for patients with visual, cognitive, and physical impairments, which was added by Lal et al [45]. Glass et al [43] provided a list of tools that can help patients throughout mHealth app use. They described, for example, trial versions during the introduction stage, written instructions during the setup stage, and direct messaging features during the follow-up stage [43].

To ensure patients receive proper training and support, medical professionals (including clinicians) should be educated to a level, where they can provide this support effectively, ultimately generating a benefit for the patient (refer to Medical Training and Curricula section); this was reported by 4 studies [36,41,42,49]. As 1 study pointed out, this contributes to patients increasing their digital literacy and ensuring they have the knowledge, skills, and confidence to participate in the use of mHealth apps (and all other forms of digital care) [36]. Different suggestions were made throughout the studies about who should deliver this support. It ranged from unspecified “support staff” [36] to clinicians and general practitioners [42]. The studies also described different ways of educating medical professionals. Gerlinger et al [42] laid out a 2-hour training session combined with the distribution of information material. Others suggested training through “demonstrations or video” [41]. All these approaches aimed at increasing the level of mental mHealth competencies on the side of practitioners to improve their ability to support the app-using patient [49].

## Discussion

### Principal Findings

This scoping review maps the evidence regarding the integration and implementation of mHealth apps for mental disorders into the health care context. On the one hand, different studies and reviews have identified problems surrounding mHealth apps (eg, [15,55,56]). In their scoping review, Giebel et al [15] identified problems regarding the implementation, among others, in 17 studies. They described “organizational barriers, such as lack of capacity or preparedness of healthcare systems and reimbursement structures,” and the “successful transfer into clinical practice was seen as a problem” [15,57-59]. On the other hand, the number of reported symptoms of mental disorders in “Organization for Economic Co-operation and Development” - countries increased during the COVID-19 pandemic. This increase slowed down toward the end of the pandemic, but levels of reported symptoms remain at least 20% higher, in some countries even doubling the prepandemic rates [60]. The American Psychological Association suggested leveraging technology such as mHealth apps to meet the rising demand [61]. This highlights the importance of a review aiming to identify concepts and studies for integration and implementation into outpatient mental health care systems. As our approach was to analyze this knowledge gap and identify and map the key concepts in the literature, we decided to use the scoping review method proposed by Arksey and O’Malley [17]. To the best of our knowledge, this is the first review drawing up the international evidence surrounding the described topic.

As presented in the General Requirements section, an overarching infrastructure, particularly a reliably functioning one, is necessary to ensure all processes surrounding the mHealth apps are standardized and sufficient. The creation and adaptation of such infrastructure and corresponding processes pose challenges for some countries. This may also be due to the general attitude of the involved stakeholders. In Germany, digitalization and the increasing use of technology in the health care system are often met with skepticism and resistance. This may also be the case due to an often-changing course of public health policy and many “slip-ups” and delays in execution of new initiatives [62,63], creating an insufficient environment for innovation [64].

A challenging aspect of mHealth apps, both in general and with their integration, is their distinct nature as digital health care solutions. On the one hand, there are similarities on a regulatory basis to medication and pharmaceuticals, such as, for example, the prescription process and the use (mostly) outside of the health care facility. On the other hand, mHealth apps are often digital equivalents of health care services normally delivered by personnel. Many of the mHealth apps (DiGA) for mental disorders in Germany are based on the principles of cognitive behavioral therapy [9], which is usually delivered by psychotherapists in the outpatient setting. This raises the question of how to optimally integrate mHealth apps and consequently, when they should be used. On the one hand, they can be used as a prescribable health care service, in combination with and monitored alongside regular therapy provided by medical professionals. On the other hand, they can be seen as—with a new perspective on service delivery—stand-alone solutions delivering therapy procedures to patients. Regarding the latter, the human component cannot be neglected. The lack of human interaction is described as problematic [15], which is relevant in the context of mHealth apps for mental disorders. This is expressed in low levels of empathy provided by mHealth apps and the reduction of complex human interactions within apps, affecting use and adherence of patients [57,65,66]. In countries such as Germany with problems in access and distribution of psychotherapeutic care [4,67,68], mHealth apps may (partly) provide relief, but possible effects of the lack of human interaction (eg, illness due to loneliness or worsening of the disease) have to be kept in mind.

The level of involvement of medical professionals and the support required for patients may also stand in relation to their digital literacy. The use of mHealth apps with involvement of a medical professional for patients with low digital literacy may be just as beneficial as the use of mHealth apps by patients with high digital literacy alone. DiGA manufacturers in Germany reported that their approach shows effectiveness with and without guided use as well as self-guided use [69,70]. Therefore, the individual skills and literacies of the patients and indication-related aspects have to be kept in mind when integrating or implementing DiGA.

One major problem of this review was the heterogeneity of health care systems of the included mHealth apps and corresponding studies. We included, for example, studies from the United States, on the one side, with a largely privately organized health care system [34,36,37,40,41,43,44,46-48], and on the other side, articles from Germany, with a large SHI system [39,42,49,50]. The integration of an mHealth app into a private health care organization and corresponding conclusions cannot necessarily be transferred to the integration into a health care system where 90% of the population is insured within the public SHI system [71]. Large publicly (statutory) organized insurance systems are less agile when having to adopt to structures and processes because they are bound to legislation and health policies for change, as, for example, in Germany [9].

This review showed that the integration of mHealth apps into health care settings is often associated with direct involvement of clinicians or other medical professionals. At the same time, access and distribution problems—as described earlier—already exist in mental health care in Germany, for example, having to wait up to months [4,67,68]. There is also a shortage of available personnel, particularly in medical support roles [72]. As our goal was to identify concepts for integration into health care systems and explore how they may be adapted and transferred to the German health care system, we recognize the need to create time for medical professionals when providing services such as guided app use. At the same time, with the currently available workforce and the arising workload, this does not seem bearable or feasible. A solution might be possible through blended care models. Rethinking care processes and including traditional care elements and mHealth apps at the same time [47,49] may provide a solution to integrate mHealth apps into the care context and at the same time attend to current problems in mental health care, as described earlier.

The review also showed that integration of mHealth apps is primarily discussed within the context of ongoing treatments or diseases (usually) requiring treatment by a medical professional (eg, PTSD) [47]. Bridging back to the German health care context, there are also DiGA approved for the treatment of, for example, nicotine addiction [9], which is often not treated through outpatient psychotherapeutic, psychiatric, or general practitioners’ care. This raises the question of whether the requirements for integrating mHealth apps for such indications differ from those for indications with more involvement of medical professionals. This may also be relevant for patients with lower severity of certain diseases or patients not currently needing “real, personal” treatment but still looking for help.

### Limitations

This scoping review aims to identify concepts for the integration and implementation of mHealth apps into health care contexts. Therefore, we designed a relatively broad search strategy to grasp all relevant evidence. Nevertheless, although we used an explorative search to map all relevant search terms, some studies may not be included in our results.

We also focused on the following 3 databases: PubMed, Embase, and PsycINFO. Studies not indexed in these 3 databases may have not been identified within our review. We minimized this potential limitation by performing a targeted search, as described above (refer to Step 6: Targeted Literature Search section). The fast-evolving nature of the digital age also poses the risk of relevant new studies being published after the literature is extracted from the databases.

Our study is also limited to published concepts of integration. Other concepts, which are currently used within mental health care contexts but have not been published yet, could not be identified within our scoping review.

One methodological problem was the definition of mHealth apps. We could not identify a consensus in the literature and therefore operationalized our definition guided by Kasperbauer and Wright [22], Möllers and Weider [23], Shabir et al [24], and the official definition of DiGA in § 33a SGB V. All articles not matching this specific definition were excluded from our review.

As we limited the date of publication from 2007 to 2022, we excluded all studies published before 2007. We estimate that the number of relevant studies we could have missed before 2007 is minimal because 2007 was the year the first smartphone and the concept of mobile apps were introduced globally [26].

### Conclusions

Digital technologies are becoming an increasingly integral part of everyday life, including in the health care sector. According to the providers of mHealth apps, they are already playing an important role in improving patients’ health and treatment outcomes. These positive effects have been shown in scientific studies [73].

Nevertheless, their integration into existing care settings still poses a lot of barriers. To address these problems, we identified a range of requirements and concepts on how mHealth apps can be integrated and implemented into outpatient mental health care settings. As Germany already has regulations for reimbursable mHealth apps in place, some of these solutions were already implemented, for example, the evaluation and directories. Others were lacking transferability due to certain circumstances in the German health care system (eg, staff shortage and workload).

However, more use of medical professionals through blended care models, improving information deficits, as well as better training and support of medical professionals and patients provide promising concepts for better integration of mHealth apps into health care settings.

This review expands on existing literature by being the first, to our knowledge, to combine mHealth apps as a health care service reimbursed by SHI with concepts for integration into mental health care. The evidence provides insights on superordinate regulatory aspects as well as concepts for the integration into everyday care. Therefore, this scoping review may provide guidance for policy makers aiming to improve (mental) health care through integration efforts. From a methodological standpoint, our approach of defining mHealth apps based on DiGA may be useful for other researchers pursuing similar research topics.

As this scoping review is part of a larger research project focusing on realizing the potential of mHealth apps (more specifically, DiGA) in outpatient mental health care within the German health care setting, the identified concepts for integration of mHealth apps are used as an evidence base for upcoming project modules, such as interviews and a survey.

## Appendix

Multimedia Appendix 1 Search strategy.

Multimedia Appendix 2 Overview of the included studies.

Multimedia Appendix 3 Overview of the excluded studies.

Multimedia Appendix 4 Overview of the categories with descriptions.

Multimedia Appendix 5 PRISMA-ScR checklist.

## Abbreviations

BfArM: Federal Institute for Drugs and Medical Devices
DiGA: Digitale Gesundheitsanwendungen
MeSH: Medical Subject Headings
mHealth: mobile health
PCC: population, concept, and context
PRISMA-ScR: Preferred Reporting Items for Systematic Reviews and Meta-Analyses Extension for Scoping Reviews
PTSD: posttraumatic stress disorder
SHI: statutory health insurance
