# Supplementary material for: Concepts for the Integration and Implementation of mHealth Apps for Patients With Mental Disorders: Scoping Review
Source: J Med Internet Res. 2025 Sep 8;27:e66340. doi: 10.2196/66340 (PMC12455162; doi:10.2196/66340)
Supplement: Multimedia Appendix 1 [file jmir_v27i1e66340_app1.docx]

**Search strategy (PubMed) and Coding Scheme (MAXQDA)**

1. **Search strategy**

**Patients with mental disorders (Population)**

(mental disorders [Mesh Major Topic] OR mental disorder* [TIAB] OR mental illness* [TIAB] OR mental disease* [TIAB] or psychiatric disease* [TIAB] OR psychiatric disorder* [TIAB] OR mental health [MeSH Major Topic] OR mental health [TIAB])

**Integration into care (Context)**

(implementation* [TIAB] OR integration* [TIAB] OR ‘introduction of’ [TIAB] OR ‘introduction to’ [TIAB] OR adoption [TIAB] OR insertion [TIAB] OR framework [TIAB] OR guideline* [TIAB] OR strateg* [TIAB])

**mHealth apps (Concept)**

(mobile applications [MeSH Major Topic] OR digital health app* [TIAB] OR mHealth [TIAB] OR eHealth [TIAB] OR healthcare app* [TIAB] OR health care app* [TIAB] OR mobile health [TIAB] OR health app* [TIAB] OR virtual care [TIAB] OR digital intervention [TIAB] OR web app* [TIAB] OR mobile app* [TIAB] OR smartphone [TIAB] OR smart phone [TIAB] OR mobile phone [TIAB] OR android [TIAB] OR iphone [TIAB] OR browser [TIAB])

**Search strategy 1 (complete)**

(mobile applications [MeSH Major Topic] OR digital health app* [TIAB] OR mHealth [TIAB] OR eHealth [TIAB] OR healthcare app* [TIAB] OR health care app* [TIAB] OR mobile health [TIAB] OR health app* [TIAB] OR virtual care [TIAB] OR digital intervention [TIAB] OR web app* [TIAB] OR mobile app* [TIAB] OR smartphone [TIAB] OR smart phone [TIAB] OR mobile phone [TIAB] OR android [TIAB] OR iphone [TIAB] OR browser [TIAB]) **AND** (implementation* [TIAB] OR integration* [TIAB] OR ‘introduction of’ [TIAB] OR ‘introduction to’ [TIAB] OR adoption [TIAB] OR insertion [TIAB] OR framework [TIAB] OR guideline* [TIAB] OR strateg* [TIAB]) **AND (mental disorders [Mesh Major Topic] OR mental disorder* [TIAB] OR mental illness* [TIAB] OR mental disease* [TIAB] OR mental health [MeSH Major Topic] OR mental health [TIAB] OR psychiatric disease* [TIAB] OR psychiatric disorder* [TIAB])**

**Table 1:** Search strategy and number of hits (PubMed)

| Database | PubMed | |
| --- | --- | --- |
| Platform | Pubmed.gov | |
| Date of search | 25.01.2023 | |
|  |  | |
| # | **Search Term** | **Hits** |
| 1a | mobile applications [MeSH Major Topic] | 8,943 |
| 1b | web app* [TIAB] | 3,737 |
| 1c | mobile app* [TIAB] | 9,280 |
| 1d | digital health app* [TIAB] | 235 |
| 1e | mHealth [TIAB] | 9,043 |
| 1f | eHealth [TIAB] | 9,123 |
| 1g | virtual care [TIAB] | 1,051 |
| 1h | healthcare app* [TIAB] | 1,441 |
| 1i | health care app* [TIAB] | 830 |
| 1j | mobile health [TIAB] | 7,909 |
| 1k | health app* [TIAB] | 9,296 |
| 1l | digital intervention [TIAB] | 642 |
| 1m | smartphone [TIAB] | 18,209 |
| 1n | smart phone [TIAB] | 1,048 |
| 1o | mobile phone [TIAB] | 10,509 |
| 1p | android [TIAB] | 3,641 |
| 1q | iphone [TIAB] | 1,129 |
| 1r | browser [TIAB] | 4,014 |
|  |  |  |
| 1s | **1a OR 1b OR 1c OR 1d OR 1e OR 1f OR 1g OR 1h OR 1i OR 1j OR 1k OR 1l OR 1m OR 1n OR 1o OR 1p OR 1q OR 1r** | **70,100** |
|  |  |  |
| 2a | implementation* [TIAB] | 339,085 |
| 2b | integration* [TIAB] | 217,935 |
| 2c | ‘introduction of’ [TIAB] | 176,214 |
| 2d | ‘introduction to’ [TIAB] | 30,172 |
| 2e | adoption [TIAB] | 68,621 |
| 2f | insertion [TIAB] | 169,620 |
| 2g | framework [TIAB] | 348,214 |
| 2h | guideline* [TIAB] | 457,067 |
| 2i | strateg* [TIAB] | 1,444,393 |
|  |  |  |
| 2j | **2a OR 2b OR 2c OR 2d OR 2e OR 2f OR 2g OR 2h OR 2i** | **2,903,119** |
|  |  |  |
| 3a | mental disorders [Mesh Major Topic] | 1,193,510 |
| 3b | mental disorder* [TIAB] | 56,785 |
| 3c | mental illness* [TIAB] | 39,389 |
| 3d | mental disease* [TIAB] | 3,970 |
| 3e | psychiatric disease* [TIAB] | 6,746 |
| 3f | psychiatric disorder* [TIAB] | 50,112 |
| 3g | mental health [MeSH Major Topic] | 30,968 |
| 3h | mental health [TIAB] | 213,694 |
|  |  |  |
| 3i | **3a OR 3b OR 3c OR 3d OR 3e OR 3f OR 3g OR 3h** | **1,384,233** |
|  |  |  |
| 4 | **1s AND 2i AND 3i** | **2,290** |
|  |  |  |
| 5 | **4** AND Filters: **English, German, 2007-2022** | **2,187** |
|  |  |  |
|  |  |  |

Please contact the author for information on the search strategies used for other databases.

1. **Coding Scheme (used for evidence extraction in MAXQDA)**
2. **Study**
   1. Author
   2. Year
   3. Title
   4. Country
3. **Population**
   1. Study Population
   2. Setting
4. **Methods**
   1. Study Design
   2. Data collection
   3. Data evaluation
5. **mHealth App**
   1. Name of App
   2. Description
   3. Age
   4. Disease
   5. Scientific Evidence
6. **Concepts for integration and implementation**
   1. *General requirements*
      1. Technological considerations
      2. Organizational considerations
      3. Legal considerations
      4. Financial considerations
   2. *Concepts and Strategies*
      1. Evaluation of mHealth Apps
      2. Conceptional Basics, models etc.
      3. Regulation
      4. Public relations and information
      5. Guidelines
      6. Directories
      7. Integration of other technological components
      8. Clinical workflow and adaption
      9. Training and support of medical professionals
      10. Training and support of patients
      11. Medical Training and Curricula
      12. Adherence
