# Supplementary material for: Concepts for the Integration and Implementation of mHealth Apps for Patients With Mental Disorders: Scoping Review
Source: J Med Internet Res. 2025 Sep 8;27:e66340. doi: 10.2196/66340 (PMC12455162; doi:10.2196/66340)
Supplement: Multimedia Appendix 4 [file jmir_v27i1e66340_app4.docx]

| **Category** | **Description** | **Example** |
| --- | --- | --- |
| Financial considerations | This category includes all evidence regarding financial aspects of the integration of mHealth apps into mental healthcare, e.g., reimbursement for patients and healthcare professionals. | Funding/reimbursement by health insurance funds [Gerlinger et al. (2021)] |
| Legal considerations | This category includes legal formalities, e.g., legal definitions, laws and other judicial requirements. | Definition and borders of liability of manufacturers and healthcare providers in the context of mHealth apps [Connolly et al. (2021)] |
| Organizational considerations | Organizational considerations are aspects regarding the organizational infrastructure, including, e.g., hierarchy or responsibilities of leadership / management personnel. | Support from leadership and their engagement on an organizational level [Ford et al. (2015), Possemato et al. (2017), Silfee et al. (2021)] |
| Technological infrastructure | This category contains all technological aspects outside of the mHealth app. This includes the superordinate infrastructure (e.g., sufficient internet access) as well as necessary hardware (e.g., laptop, smartphone). | Superordinate, technological infrastructure for a safe, reliable and standardized operation of mHealth apps [Austin et al. (2020), Gerlinger et al. (2021)] |

**General requirements**

Studies cited correspond to the main article. See main article for full references.

**Concepts for integration**

| **Category** | **Description** | **Example** |
| --- | --- | --- |
| **Theoretical aspects** | |  |
| Conceptional basics | This category contains theoretical frameworks regarding the integration of mHealth apps into healthcare. This includes complete frameworks as well as singular theoretical aspects. | Consolidated Framework for Implementation Research (CFIR) [Possemato et al. (2017)] |
| **Regulatory concepts** | |  |
| Evaluation of mHealth apps | This includes all approaches and the including processes aiming at evaluating mHealth apps before they are used by patients within a defined healthcare system. Evaluations within app design and development are of secondary interest for this category. | American Psychological Association (APA) pyramid model, Elight and the Mobile App Rating Scale [Chan et al. (2017b)] |
| Medical training and curricula | This category contains approaches of integrating mHealth apps into medical training and the corresponding curricula. | Medical training of medical providers and further education throughout the professional career, with funding and reimbursement [Gerlinger et al. (2021)] |
| Directories | This category contains software or website driven approaches listing mHealth apps (for mental disorders). They can be regulated federally or independent, providing information for different interest groups. | DiGA-directory [Weitzel et al. (2021)] |
| **Care-related concepts** | |  |
| Adherence-improving strategies | These concepts are aiming to improve adherence of patients using mHealth apps. This also includes all therapy elements associated with the mHealth app, but not carried out by the app directly, e.g., consultations with healthcare professionals about the app. | Automated reminders [Dinkel et al. (2021), Glass et al. (2021), Kreyenbuhl et al. (2019)] |
| Clinical workflow and adoption | Concepts listed within this category are aiming to adapt (traditional) clinical workflows to better align them with mHealth apps and therefore improve their integration. | Integration of screening tools for mHealth app use into e.g., anamnesis [Possemato et al. (2017)] |
| Guided app-use | Guided app use contains different concepts, which assist patients when using mHealth apps (e.g., through blended care models). These concepts are carried out by healthcare professionals with various backgrounds (e.g., physicians, physician assistants). | CS-PTSD Coach [Possemato et al. (2017)], blended care models [Gerlinger et al. (2021)] |
| Integration of additional technical components | This category aims at additional technological components and their integration and interaction with mHealth apps. This includes the access to and transfer of the generated data. | Transfer of mHealth app generated data into electronic health records [Dinkel et al. (2021), Kreyenbuhl et al. (2019), Silfee et al. (2021)] |
| Public relations and information | Information initiatives with public recognition are summarized in this category. This contains media (printed, digital and others) and information through human interaction (e.g., public gatherings). | Information material, e.g., flyers/brochure [Fleddermann et al. (2021)] |
| Training and support of users and healthcare providers | This category includes training and support efforts aiming at healthcare providers and users’ (patients’) knowledge regarding mHealth apps. This category focuses on the training and knowledge surrounding mHealth apps, rather than guided use (for this topic see Guided app-use). | Introduction of the mHealth app and important tasks by healthcare provider [Chan et al. (2017a)] |

Studies cited correspond to the main article. See main article for full references.
